# Physiological responses to affiliation during conversation: Comparing neurotypical males and males with Asperger syndrome

**DOI:** 10.1371/journal.pone.0222084

**Published:** 2019-09-18

**Authors:** Melisa Stevanovic, Pentti Henttonen, Emmi Koskinen, Anssi Peräkylä, Taina Nieminen von-Wendt, Elina Sihvola, Pekka Tani, Niklas Ravaja, Mikko Sams

**Affiliations:** 1 Faculty of Social Sciences, University of Helsinki, Helsinki, Finland; 2 Faculty of Medicine, University of Helsinki, Helsinki, Finland; 3 Neuropsychiatric Rehabilitation and Medical Center, NeuroMental, Helsinki, Finland; 4 Helsinki University Hospital, Helsinki, Finland; 5 Department of Neuroscience and Biomedical Engineering, Department of Computer Science, Aalto University, Espoo, Finland; Birkbeck University of London, UNITED KINGDOM

## Abstract

We examined the emotional and psychophysiological underpinnings of social interaction in the context of autism spectrum disorder, more specifically, involving participants diagnosed with Asperger syndrome (AS). We recorded participants’ autonomic nervous system (ANS) activation (electrodermal activity, heart rate, and heart rate variability) and facial muscle activation during conversations in two different types of male dyads: (1) ten dyads where one participant has been diagnosed with AS (AS/NT dyads) and (2) nine dyads where both participants are neurotypical (NT/NT dyads). Afterwards, three independent raters assessed continuously each participant’s affiliative and dominant behaviors during the first and last 10 minutes of the conversations. The relationship between the assessed data and ANS responses was examined. We found that, in the NT/NT dyads, a high level of affiliation displayed by the conversational partner calms down the participant when they are actively dominating the interaction. In contrast, when the participants themselves expressed affiliation, their psychophysiological responses indicated increase in arousal, which suggests that the giving of affiliation is physiologically “hard work.” The affiliation-related ANS responses were similar in those NT participants whose conversational partner had AS, while some differences in facial muscle activation did occur in comparison to NT/NT dyads. In the AS participants, in contrast, a high level of affiliation provided by the conversational partner was associated with increase in arousal, suggesting heightened alertness and stress. As for their own affiliative behavior, the AS participants exhibited similar indicators of alertness and stress as the NT participants, but only when their own level of dominance was low. Our results increase understanding of how individuals with AS experience social interaction at the physiological level, and how this experience differs from that in NT individuals. Moreover, our results confirm and further specify our earlier results, where we proposed that affiliation involves the type of “sharing of the burden” that also reverberates in the participants’ bodies.

## Introduction

In this paper, we investigate the emotional and psychophysiological underpinnings of social interaction in the context of autism spectrum disorder—more specifically, with participants diagnosed with Asperger’s syndrome (AS) [1–3]. We compare autonomic nervous system (ANS) responses and facial muscle activations (*valence expressions*) of individuals with AS with those of neurotypical participants during self- and other-expressed affiliative behaviors.

The notion of affiliation has been widely used in the empirical social-interaction research literature (for an overview, see [4]). The term has been used to describe behaviors by which a conversational partner displays that he or she supports the affective stance expressed by the speaker, for example, in the context of storytelling [5] or troubles-talk [6]. The giving or withholding of affiliation has been shown to influence the subsequent trajectories of interaction, which happens, for example, when storyteller recycle the punchline of their story in order to pursue recipient affiliation [7–9]. Affiliation has also been found to reverberate in the participants’ bodies. In the seminal work, Peräkylä and colleagues [10] found that verbal and nonverbal displays of affiliation by a story recipient decreased the storyteller’s level of arousal. At the same time, these affiliation displays increased the story recipients’ own level of arousal, which suggests that the giving of affiliation is physiologically taxing “emotional labour” [11, 12]. In this study, we consider the influence of affiliation displays on the participants’ physiological responses throughout conversational episodes (and not merely during the moments of storytelling), while we assume that these responses are mediated by the participants’ moment-by-moment status of dominance in the conversation (and not only by the roles of the storyteller and recipient).

The larger motivation for our study arises from the enactive account of autism sketched by Hanne De Jaegher [13], who has used the term *autistic embodiment* to refer to the “particular ways in which the biology, neurophysiology, affective, and sensorimotor structures and skills of people with autism differ from those of non-autistics” [13, p. 8]. These differences have been seen to relate inextricably to how individuals on the autism spectrum make sense of the world, and this sense-making is what we seek to shed light on by looking at ANS responses of these individuals.

Individuals with autism spectrum disorders (ASD) have been found to have trouble inferring other people’s mental events [14], for example, in terms of predicting future actions [15] or reasoning backwards from observed behavior to an antecedent cause [16, 17]. Such difficulties have been found to occur frequently during the processing of dynamic stimuli or complex emotion responses [18], which has been accounted for with reference to deficits of social attention [19, 20]. As for the difficulties of emotion processing, the situation is more complex. On the one hand, some ASD related behaviors, such as gaze aversion, which inevitably complicate the interpretation of emotional responses in other people, have been described as a strategy to reduce cognitive demands in face-to-face interactions (e.g.[21]). On the other hand, neurotypical individuals have also been shown to have difficulties in interpreting the behaviors of individuals with ASD [22]. Therefore, what has been described as a “double empathy problem” [23] involves a breakdown in reciprocity and mutual understanding, which is not to be attributed only to a cognitive deficit of individuals with ASD. Thus, in considering the ways in which affiliation is both expressed and received by both neurotypical participants and participants with ASD, this study can corroborate the idea of bi-directionality of the ASD social interaction deficit.

In this study, we treat ANS responses and valence expressions as a window to how participants experience interactional event in their bodies. ANS consists of two components: sympathetic and parasympathetic, which work together to regulate physiological arousal and other body functions [24]. The sympathetic nervous system primes the body for action. The parasympathetic nervous system, then again, works to maintain homeostasis and resting state of the body. Sympathetic response in its purest form can be called the stress response and can be measured through *electrodermal activity* (EDA; indexed by increased palmar skin conductance). Parasympathetic component of the autonomic nervous system can be measured in the cyclical control of breathing over heart rate through an index of *heart rate variability* (HRV), the high frequency (HF) index. *Heart rat*e (HR) is under the control of both the sympathetic and parasympathetic branches of ANS. The presence of high frequency component (0.15–0.4 Hz) in HRV (HRV-HF) reflects less stress and higher physiological flexibility in adapting to a changing environment [25]. As for valence expressions, the activity of the orbicularis oculi muscle (EMG-OO) can be considered as in index of smiling and positive emotions, whereas activity of corrugator supercilii (EMG-CS) serves as an indication of frowning and negative emotions.

There is emerging evidence that ANS functions atypically in autism spectrum disorders and that these atypicalities may underlie or reflect the social communication difficulties associated with the condition (for an overview, see e.g., [26]). Such atypicalities include elevated basal HR [27, 28] and blunted HR response to psychosocial challenges [29, 30], as well as altered HRV indices [31–33]. The relation of EDA to socially relevant stimuli has also been demonstrated to be atypical in individuals with autism [34, 35], but the results of these studies are mixed. Hubert and colleagues [36] showed that individuals with ASD exhibited less arousal than controls when judging emotional facial expressions. In contrast, Kylliäinen and Hietanen [37] found enhanced arousal to direct eye contact in children with autism. Regarding EDA, there are also some studies that involve a more genuine interactional setting [38, 39], but there are no previous studies that measure psychophysiology in ASD in naturally unfolding interaction with relation to the moment-by-moment dynamics within the discussion. This study seeks to fill this gap in the literature.

To unravel the concrete features of conversation that underlie its emotional and psychophysiological outcomes in both neurotypical (NT) and Asperger (AS) participants, we will resort to interpersonal theory [40–42]. The theory postulates that the most important variation in interpersonal behavior occurs along just two dimensions: friendliness *vs* hostility, and dominance *vs* submissiveness [42–44]. We will henceforth refer to these two dimensions as *affiliation* and *dominance*. The notion of the *interpersonal circumplex* refers to the idea that different characterizations of interpersonal behavior, such as shyness or assertiveness, form a circular arrangement [41, 43, 45, 46]. The circle of variables is organized around two orthogonal axes: affiliation and dominance, which form a two-dimensional Cartesian plane.

Traditionally, the idea of interpersonal dynamics has been used to characterize the relatively static interpersonal styles of the two persons in a dyad [47–49], but later it has also been applied to the description of moment-to-moment changes in the participants’ behaviors during dyadic interaction [50, 51]. The method used in these studies is based on the assumption that the two-dimensional circumplex structure of interpersonal behaviors is intuitively accessible to raters [45], and it consists of naïve observers watching episodes of video-recorded social interaction on a computer monitor, focusing their attention on one participant at a time and, using a computer joystick, assessing continuously the levels of that participant’s dominance and affiliation behavior.

In this study, we make use of the method above, while beings guided by a number of predictions and hypotheses. First, seeking to replicate the findings by Peräkylä and colleagues [10], we consider the effect of the co-participants’ displays of affiliation on the participants’ physiological responses. Regarding the neurotypical participants, we presume that the affiliation provided by the conversational partner is associated with a less stressful physiological state and that this will show particularly during high levels of self-expressed dominance—that is, when the participant himself actively contributes to the ongoing conversation (as when telling a story). Specifically, we hypothesize that (1) partner affiliation will decrease the participants’ EDA and HR, as well as their EMG-CS (frown), and increase their HRV-HF and EMG-OO (smile).

Second, we consider the effects of self-expressed affiliation on the participants’ physiological responses. Relying on the notion of “emotional labor” by Hochschild [11, 12] and the results of the storytelling study by Peräkylä and colleagues [10] we assume that, in the neurotypical participants, the providing of affiliation constitutes physiologically taxing interactional work. We thus hypothesize that (2) self-expressed affiliation will decrease the participants’ HRV-HF and increases their EDA and HR, as well as the level of their valence expressions (both EMG-OO and EMG-CS).

Third, we consider the physiological responses to affiliation in AS participants. Based on the idea that individuals with ASD are susceptible to cognitive overload and on earlier findings that suggest a correspondence between increased ANS activity and cognitive challenges in the production of affiliation [52], we maintain that AS participants experience self-expressed affiliation as particularly taxing due to the cognitive load of interpreting emotional cues. Hence, we hypothesize that (3) self-expressed affiliation will generate larger effects in ANS activity in AS participants, compared to neurotypical participants. In addition, based on earlier suggestions that it might be relaxing for the AS participants to talk about their own specific interests [53, p. 54], we assume that the AS participants’ psychological responses might be reduced when they are dominating the interaction. Thus, we hypothesize that (4) self-expressed dominance by the AS participants will lead to differentiated effects in their ANS activity during high *vs* low dominance segments.

Finally, given that conversational social interaction is always a collaborative endeavor, we expect that in AS/NT dyads both neurotypical participants and participants with AS will show atypical psychophysiological responses. More specifically, we presume that the displays of affiliation provided by an AS participant will cause higher ANS responses in their neurotypical co-participants than the displays of affiliation that neurotypical participants provide to each other. In other words, the affiliation provided by the AS participant might *not* “calm down” the neurotypical participant. So, we hypothesize that (5) partner-affiliation leads to diminished or opposite effects in ANS activity in neurotypical participants conversing with an AS participant, compared to the effects observable in conversations between two neurotypical participants. Furthermore, reflecting the double empathy problem described earlier, we suggest that a similar pattern can be found also in the AS participants’ responses to the displays of affiliation provided by the NT participants. Hence, our final hypothesis is that (6) the displays of affiliation provided by the neurotypical participants will cause elevated ANS responses in the AS participants conversing with them.

## Materials and methods

### Participants

The video-recorded interaction material used in this study consisted of 19 Finnish face-to-face conversations between previously unacquainted dyads, who had been instructed to discuss happy events and losses in their lives. The discussions were free to unfold in any shape or form and the participants also talked about mundane topics, such as studies and hobbies. The dyads consisted of 10 AS/NT dyads (i.e., one AS participant and one NT participant) and 9 NT/NT dyads. All participants were Finnish speaking males, with ages ranging from 19 to 40. To control for the relative emotional salience of discussions, we asked the participants to fill in a post-conversation questionnaire where they were encouraged to list those emotionally salient topics that they had dealt with during the discussion. We found that there was an equal amount of happy/positively valenced and sad/negatively valenced topics that both NT and AS participants listed.

The AS participants were recruited from a private neuropsychiatric clinic specialized in the diagnostics of autism spectrum disorders. All participants invited to participate in the study filled the criteria of ICD-10 [54] and had been interviewed by a psychiatrist, pediatrician, and child neurologist, all with years of expertise in AS. The diagnosis had been made with standard measurements: ADI-R (Autism Diagnostic Interview-Revised), ASSQ (Autism Spectrum Screening Questionnaire), ASDI (Asperger Syndrome Diagnostic Interview), the Benton Face Recognition Test, the Face Recognition Task from the NEPSY (Neuropsychological Test Battery for Children), and along with the autism spectrum quotient (AQ; [55]), empathy quotient (EQ; [56]) and systemizing quotient (SQ; [57]) questionnaires. In addition, an experienced neuropsychologist had performed the WAIS-R (Wechsler Adult Intelligence Scale, revised [58]). Only individuals whose WAIS-R scores were in the average range were invited to participate in the study, the recruiting clinicians being committed to find us participants who would be maximally “stereotypical” representatives of their diagnostic category. After the AS participants (N = 10) had been recruited to the study, the clinic gave us the participants’ original scores from the AQ, EQ, and SQ questionnaires, so that we could compare them with the ones we were collecting from the neurotypical participants (see below). The other original scores from the diagnostic tests we have not been able to obtain, which causes limitations in understanding the detailed characteristics of the AS participants. As we recruited the AS participants to the study, none of them had an accompanying diagnosis in addition to AS or were on psychoactive or any other medication.

The neurotypical participants were recruited to the study via email lists and their neurotypical status was confirmed by using the autism-spectrum quotient (AQ), empathizing quotient (EQ) and systemizing quotient (SQ) questionnaires. AS participants had significantly higher AQ scores (F(2,31) = 33.94, p<0.001) and lower EQ scores (F(2,31) = 8.27, p<0.01) than both NT control groups. There were no observed differences between AS/NT and NT/NT controls in their AQ (p = 0.57) or EQ (p = 0.64) scores, suggesting no a priori effects on dyad composition. There were no differences between groups in general relating to SQ scores (p = 0.73) or age (p = 0.55, see Table 1).

**Table 1:** Descriptive statistics of groups.

	NT with NT		NT with AS		AS with NT	
	Mean	SD	Mean	SD	Mean	SD
Age	23.75	3.62	25.44	2.30	25.57	7.55
AQ	11.06	4.85	9.78	4.52	27.78	6.89
EQ	45.00	9.63	47.00	10.43	29.78	10.80
SQ	31.13	11.30	30.33	10.85	36.33	13.24

The NT participants conversing with the AS participants were informed about the clinical status of their co-participants, and similarly, the status of the NT participants was revealed to the AS participants. Even if keeping both participants unaware of each other’s clinical status might have been methodologically optimal, we considered it unethical and also practically impossible.

All participants were informed about the use of the data and signed a consent form. Their identity was revealed only to two members of the research group. The study and the consent procedure were approved by the Ethics Committee of the Helsinki University Central Hospital (date of the decision: 21.09.2011).

### Video-recorded interaction material and physiological measurements

The conversations took place in an acoustically shielded room where the participants were sitting in armchairs facing each other perpendicularly. The conversations were videotaped with three cameras: two facing each of the two participants, and the third giving an overall view of the situation. The conversations lasted 45–60 minutes. After 45 minutes of discussion, the experimenter asked whether the participants wanted to continue the conversation for a maximum of 15 minutes. In both AS/NT and NT/NT dyads, there were 4 dyads wanting to continue the conversation. A 5-minute baseline (sitting still) was recorded prior to conversation.

During the conversations, we recorded the participants’ ANS responses by using two synchronized Nexus 10 portable amplifiers (Mindmedia BV, Netherlands). Electrodermal activity was sampled at 32 Hz with a constant voltage of 0.5 V with Ag/AgCl electrodes placed on the palmar middle phalanges of index and middle fingers of the non-dominant hand. Electrocardiography signal was recorded with 512-Hz sampling rate using wet electrodes placed according to Lead II alternative placement guidelines [59]. Participants’ facial electromyographic responses at *Orbicularis oculi* (smile) and *Corrugator supercilii* (frown) muscles were recorded using bipolar electrode pairs with 2048-Hz sampling rate. We also measured the respiration rate of the participants with thoracic strain gauge, but the results are not reported here due to a large number of speech artifacts in the signal. We used HRV-HF to reflect vagal tone, in line with Malik [60]. Before the conversation started, a five-minute baseline was recorded, during which the participants were instructed to sit still and relax.

Ten minutes has been shown to be a sufficient length of time to pick up stable patterns of interaction [50, 61]. As one of our initial interests was to assess possible differences between the two different types of dyads in the temporal patterns of participants’ adjustment to each other’s overall levels of dominance and affiliation (see [62]), we included the two 10-minute segments from each dyadic conversation into our analysis: one from the beginning of the conversation and the other from the end of it (minutes 35–45, before the above-mentioned intervention by the experimenter).

### Assessing dominance and affiliation

We used a Logitech Extreme 3D Pro joystick (http://gaming.logitech.com/en-us/product/extreme-3d-pro-joystick; see [51]) connected to a Hewlett Packard Windows PC to assess continuously the participants’ dominant and affiliative interpersonal behaviors during interaction segments. The in-house code for the measurement was written with Matlab’s Psychophysics Toolbox extensions [63–65]. A 12 x 12 cm Cartesian plane was displayed on the computer screen. The left and right endpoints of the x-axis were labeled as maximally hostile and friendly, respectively, and the top and bottom endpoints on the y-axis as maximally dominant and submissive. Scale on both axes ranged from 0 to 65535 such that the midpoint on the Cartesian plane (the rest position of the joystick) was at (32767, 32767). The joystick position, sampled at 30 Hz, was indicated by a white dot on the computer screen. Trained observers used the joystick to evaluate the magnitude of affiliation- and dominance-related behaviors of the participants. The video of the interaction being rated appeared on the same computer screen as the joystick position, so that the observers could watch the interaction and see their current joystick position simultaneously. The video appeared within a 25 x 30 cm window, and the joystick position was always located on the same side of the screen as the participant being rated.

### Obtaining observer ratings

Three independent observers rated the behaviors of all participants. The observers were graduate students, who were unaware of the research questions and hypotheses of the study.

The observers underwent 1–2 weeks of training with the joystick device before rating the segments used in this study. The training procedure has been described in more detail elsewhere (see [62]). Essentially, we followed the training protocol developed by Sadler and colleagues [50], where the observers are instructed to move the joystick to the correct location in the Cartesian plane in response to 16 interpersonal adjectives, such as warm, trusting, passive, unsociable, indifferent, critical, assertive and outgoing, and a set of 21 one-sentence-long verbal descriptions of interactional events, such as “The participant poses challenging questions” (maximally dominant) or “The participant provides friendly and encouraging backchannelling responses to his/her co-participant’s telling” (maximally affiliative; for the entire protocol, see S2 Appendix). After the observers have successfully completed three offline exercises, they start to practice the “online” rating of the interaction data, with the trainer monitoring each observer’s performance and discussing any problems or questions as they arise.

After the observers had finished their training, they began rating the interaction segments used in the current study. Since there were two participants and two segments to rate, each observer completed altogether 76 (19x2x2) separate 10-minute rating tasks (12 h 40 min in total). These segments were presented differently for each observer in a pseudorandomized order where we controlled that two participants from the same dyad and interaction segment were never rated consecutively.

Each 10-min rating task began with the observer pressing the start button on the joystick and starting the video. Then, the observer provided a continuous rating of the target person in the two-dimensional space: dominance-submission and hostility-friendliness. We refer to these two dimensions as affiliation (x-axis) and dominance (y-axis).

The inter-observer reliability was tested for all data and among all three raters with Cronbach’s α [66]. For affiliation α was 0.75 (acceptable) for participants in NT/NT dyads, 0.90 (good) for AS participants, and 0.90 for their NT co-participants. For dominance α was 0.95 (excellent) for participants in NT/NT dyads, 0.90 for AS participants, and 0.95 for their NT co-participants [67, 68]. Thus, overall, the indices of affiliation and dominance were highly reliable.

### Data processing

All data preprocessing was conducted using Matlab 9.3. The continuous ratings of the three observers were averaged at each time point to obtain time series for levels of affiliation and dominance. The resulting time series had a duration of 600 seconds. As we have reported elsewhere [62], the participants’ overall levels of affiliation and dominance were relatively similar in both NT and AS participants. Furthermore, as predicted by the interpersonal theory [43, 48–50], the participants’ and their co-participants’ mean affiliation scores were positively correlated across the whole data set (r = 0.37, SD = 0.14, p<0.001). Likewise, in all dyads, the participants’ high mean levels of dominance predicted low mean level of dominance in their co-participants (r = -0.82, SD = 0.10, p<0.001).

The analysis of affiliative and dominant behavior *per se* was then followed by the analysis of the physiological responses associated with these behaviors. EDA data was downsampled from 128 Hz to 4 Hz and visually inspected for artifacts (movement etc.). Sections containing artefacts were removed. ECG data was filtered and r-peaks were detected using the ECGLab toolbox [69] and visually inspected for ectopic beats and abnormal data. Continuous 4-Hz inter-beat-interval data was interpolated from beat times and converted into beats per minute (bpm) units. Facial EMG data was filtered using 20 Hz (highpass) and 200 Hz (lowpass) 3rd order Butterworth filters, rectified and resampled to 4 Hz using RMS values.

**Fig 1:**
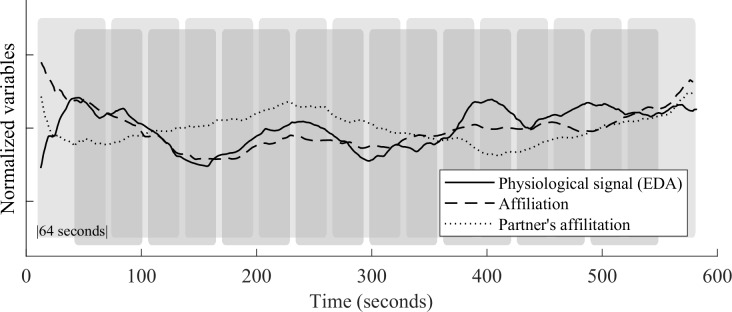
Data segmentation. Mean values of measured variables were computed for 64-second segments with 50% overlap. EDA data from an AS participant during the first 10 minutes from the start of the conversation are shown, along with data from his own and his partner’s affiliative behavior.

Each participant’s data was divided into 64-second segments with 50% overlap, resulting in a total of 34 segments (17 in beginning and ending 10 minutes). For every segment, mean values of affiliation, dominance, electrodermal activity, heart rate and electromyographic signals were calculated. Additionally, Welch’s method, smoothed with 256-point Hanning windows was used to calculate the normalized portion of energy from 0.15–0.4 Hz band to form an index of high frequency component of heart rate variability (HRV-HF). Segment length was chosen on the basis of one minute being the minimum time period for obtaining reliable HRV-HF parameters [25]. Segmentation of the data is illustrated in Fig 1.

### Analyses

Statistical analysis was done using Mixed Models procedure of SPSS 24. A linear over-time actor-partner interdependence model [70] was run separately for each dependent variable (EDA, heart rate, HRV-HF) and for different dyad types (NT/NT and AS/NT). Time of a segment, self-expressed (actor) affiliation, received (partner) affiliation and baseline mean of dependent variable were included as fixed effects. Time was also included as a random factor. Repeated effects covariance structure was modelled using *dyad* as subject variable and *role* as the repeated variable. In order to achieve suitable model specification, NT/NT (indistinguishable) and AS/NT (distinguishable) dyads were analyzed separately. Covariance structure for NT/NT dyads was CSH (Heterogeneous compound symmetry) and for AS/NT dyads CS (Compound symmetry). In case of AS/NT dyads, the effect of role along with its interactions with other fixed effects were also estimated. In addition, augmented models were run that estimated the main effects during high (above mean) and low (below mean) segments of dominance which was aggregated from continuous to categorical variable. Using a categorical variable here was justified by our main focus being on the participants’ physiological responses to *affiliation*, while we assumed, on theoretical grounds, that such responses might be sensitive to whether a participant dominates the conversation or not (cf. the roles of the storyteller and the story recipient [10]). In addition, the choice was necessitated by model simplification, where including interaction by two continuous variables would have led to interpretative difficulties of the results. The analyses were run twice, with and without dominance included as a fixed effect. For reasons of parsimony, our main analysis relies on the latter models, although we shortly also discuss the results from the former models. Reported results are standardized beta coefficients (standardized variables) for continuous actor and partner effects of affiliation predicting the dependent variable. To control for type I error due to multiple models run, significance values of all 90 estimated coefficients were adjusted using the false discovery rate procedure [71].

## Results

We will report the results testing our hypotheses by detailing the effects for the participants and their co-participants in separate sections. We will henceforth refer to the participant as *actor*, and the co-participant as *partner*. The standardized beta coefficients (standardized variables) for continuous actor and partner effects of affiliation predicting the physiological responses are reported in Table 2 (see also S1 Appendix for additional figures). Regarding other parameters, significant effects of time were observed only in heart rates, which became decelerated during both AS/NT and NT/NT conversations. AS participants (β = -0.14, SE = 0.04, t(7.94) = -3.60, p<0.01) and NT-NT participants (β = -0.13, SE = 0.03, t(6.01) = -4.03, p<0.01) had similar magnitudes of deceleration. In NT participants conversing with AS participants the effect was similar but not significant (β = -0.09, SE = 0.05, t(4.54) = -1.71 p = 0.16). Talking time during the both 10-minute phases differed between participant groups (F(2,35) = 3.66, p<0.05). AS participants (M = 435.04, SD = 89.92) talked more than NT-NT participants (M = 345.83, SD = 93.42, p = <0.05), but not significantly more than their own NT partners (M = 383.58, SD = 52.64, p = 0.18).

**Table 2:** Effects of affiliation of Actor and Partner on participant’s physiological activity in NT/NT, AS/NT, and AS/NT dyads during high and low dominance of the actor. Effect estimates are standardized betas (see Materials and methods).

		NT with NT		NT with AS		AS with NT	
		Actor	Partner	Actor	Partner	Actor	Partner
**EDA**	High dominance	0.09*	-0.07†	0.01	-0.06**	-0.03	0.10**
	Low dominance	0.10**	-0.03	-0.02	-0.05†	0.04*	0.06*
	Overall	0.10**	-0.05	0.00	-0.06**	0.02	0.06**
**HR**	High dominance	0.06†	0.01	0.09***	-0.05†	0.00	0.04
	Low dominance	0.08*	0.02	0.12**	-0.09**	-0.03†	0.04*
	Overall	0.06*	0.02	0.10***	-0.06**	-0.02	0.04*
**HRV-HF**	High dominance	-0.25*	0.19†	-0.07	-0.09	0.07	0.18
	Low dominance	-0.2*	-0.04	0.08	-0.06	0.07	0.00
	Overall	-0.26**	0.07	-0.05	-0.06	0.08	0.05
**EMG-OO**	High dominance	0.47***	0.18†	0.16*	0.03	0.08	0.05
	Low dominance	0.34**	0.25*	0.43***	-0.1	0.06	0.28***
	Overall	0.42***	0.22**	0.23***	0.01	0.05	0.23***
**EMG-CS**	High dominance	0.07†	-0.12**	0.02	0.00	0.14	0.02
	Low dominance	0.07†	-0.08*	0.09*	-0.02	-0.12†	0.12
	Overall	0.07*	-0.10***	0.04	0.00	-0.06	0.09

***) p < .001

**) p < .01

*) p < .05

†) p < .10

In addition to the analysis presented below, we run our model with dominance included as a fixed effect. Dominance was found to moderate most parameters of physiological activity: decreasing EDA (β = -0.04, SE = 0.02, F(1,424.15) = 2.72, trend), HR (β = -0.09, SE = 0.02, F(1,230.34) = 30.31, p<0.001), EMG-OO (β = -0.16, SE = 0.03, F(1,130.87) = 30.77, p<0.001) and EMG-CS (β = -0.13, SE = 0.02, F(1,246.70) = 43.351, p<0.001) and increasing HRV-HF (β = 0.13, SE = 0.07, F(1,262.74) = 4.19, p<0.05) in the NT/NT dyads. In the AS/NT dyads, similar main effects were found for EDA (β = -0.05, SE = 0.01, F(1,328.57) = 22.35, p<0.001), HR (β = -0.11, SE = 0.01, F(1,322.66) = 136.27, p<0.001), EMG-CS (β = -0.27, SE = 0.03, F(1,309.29) = 104.12, p<0.001) and EMG-OO (β = -0.11, SE = 0.01, F(1,476.64) = 37.71, p<0.001). Interaction effects with role were observed in HR (F(1,354.20) = 13.29, p<0.001), EMG-OO (F(1,327.62) = 8.97, p<0.01) and EMG-CS (F(1,526.70) = 3.32, trend), indicating that the moderating effect was greater within the AS participants. This overall “calming” effect of dominance will be below interpreted with reference to one of the main results of the study (the one according to which the providing of affiliation for another person is physiologically taxing interactional work, in which case dominance enables a participant to momentarily relax in this respect). Otherwise, with reference to affiliation, which constitutes the main focus of our study, the results obtained by using this model were mainly the same as in the model without dominance included as a fixed effect. The following analysis will therefore be based on the simpler model.

### Actor effects

Hypotheses (2), (5) and (6) were posited regarding actor effects. In the NT/NT dyads, the level of affiliation displayed by the actor correlated with physiological activity (see Table 2). Regarding hypothesis (2), a positive overall correlation (irrespective of dominance) was significant for EDA (β = 0.10, SE = 0.03, t(308.93) = 3.76, p<0.01) and HR (β = 0.06, SE = 0.02, t(312.31) = 2.89, p<0.05). Correlation was negative for the HRV-HF (β = -0.26, SE = 0.07, t(309.40)-3.64, p<0.01). The results for the NT participants in AS/NT dyads were similar for HR (β = 0.10, SE = 0.02, t(314.12) = 4.36, p<0.001). However, there were no statistically significant relationships between affiliation and EDA (β = -0.00, SE = 0.02) and HRV-HF (β = -0.05, SE = 0.06). In sum, there appears to be a heightened level of possibly stress-related physiological activation when the NT actors display high levels of affiliation.

In NT participants, affiliation was associated with significant actor effects for valence expressions, further supporting hypothesis (2) In the NT/NT dyads, self-expressed affiliation was associated with increased activation of both EMG-OO (smile) (β = 0.42, SE = 0.06, t(296.95) = 6.44, p<0.001) and EMG-CS (frown) (β = 0.07, SE = 0.03, t(353.86) = 2.834, p<0.10). Also for the NT participants in the AS/NT dyads, there was increased activation of EMG-OO (smile) (β = 0.23, SE = 0.06, t(290.13) = 3.97, p<0.001) and EMG-CS (frown) but the latter only during low dominance (β = 0.09, SE = 0.03, t(205.75) = 2.69, p<0.01).

In order to investigate differences between NT and AS subjects with reference to hypotheses (3) and (4), AS participants were observed to have idiosyncratic physiological response patterns in actor effects (see Table 2). Actor effects were mostly non-significant and thus not larger than in the NT group, invalidating hypothesis (3). The only statistically significant effect was the relationship between heightened level of self-expressed affiliation and stronger EDA during low dominance (β = 0.04, SE = 0.02, t(298.60) = 2.39, p<0.05). Similarly, AS participants had no significant actor effects regarding valence expressions. Observed mediation of partner effect by dominance in AS participants provides some support for hypothesis (4).

### Partner effects

Hypotheses (1), (5) and (6) were investigated by looking at partner effects. Regarding the presence of main partner effect concerning hypothesis (1), we observed that, in the NT/NT dyads, actor’s physiological activation was connected to the level of affiliation provided by his partner. This effect, however, was dependent on the actors’ own level of dominance. During those segments where the actor dominated the conversation, the actor’s EDA was the weaker when the partner’s level of affiliation was higher (β = -0.07, SE = 0.03, t(346.82) = -2.10, p<0.10). In line with the effects of partner affiliation on EDA, partner affiliation was also associated with increased HRV-HF, when the actor’s own level of dominance was high (β = 0.18, SE = 0.08, t(369.87) = 2.02, p = 0.10). There was no significant effect during low dominance (or independent of dominance). For valence expressions, NT participants in the NT/NT dyads exhibited significant partner effects. Partner affiliation was associated with increased EMG-OO (smile) (β = 0.22, SE = 0.06, t(191.51) = 3.76, p<0.01) and decreased EMG-CS (frown) (β = -0.10, SE = 0.02, t(325.27) = -4.15, p<0.001) activation.

Partner effects were then compared between participant groups. In AS/NT dyads, the physiological responses of NT participants to partner affiliation were relatively similar to those observed in NT/NT dyads, thus disproving hypothesis (5). There was a statistically significant negative effect of partner affiliation for EDA (β = -0.06, SE = 0.02, t(328.29) = -3.59, p<0.01) and for HR (β = -0.06, SE = 0.02, t(311.33) = -3.10, p<0.01). As posited by hypothesis (6), however, in the AS participants in AS/NT dyads, the physiological responses to partner affiliation were opposite to those observed in the NT participants. EDA showed a positive main effect of partner affiliation (β = 0.06, SE = 0.02, t(300.61) = 3.071, p<0.01), as did also HR β = 0.04, SE = 0.02, t(299.04) = 2.92, p<0.05). Regarding valence expressions (EMG-OO and EMG-CS), NT participants in the AS/NT dyads did not exhibit any partner effects. AS participants, however, displayed a positive partner effect on EMG-OO (β = 0.23, SE = 0.05, t(220.69) = 4.32, p<0.001). When considering dominance as a mediating factor, the effect was statistically significant when the AS participants’ own level of dominance was low (β = 0.28, SE = 0.06, t(232.25) = 4.98, p<0.001), but not when their own level of dominance was high.

### Illustration: The effect of partner affiliation as EDA change

**Fig 2:**
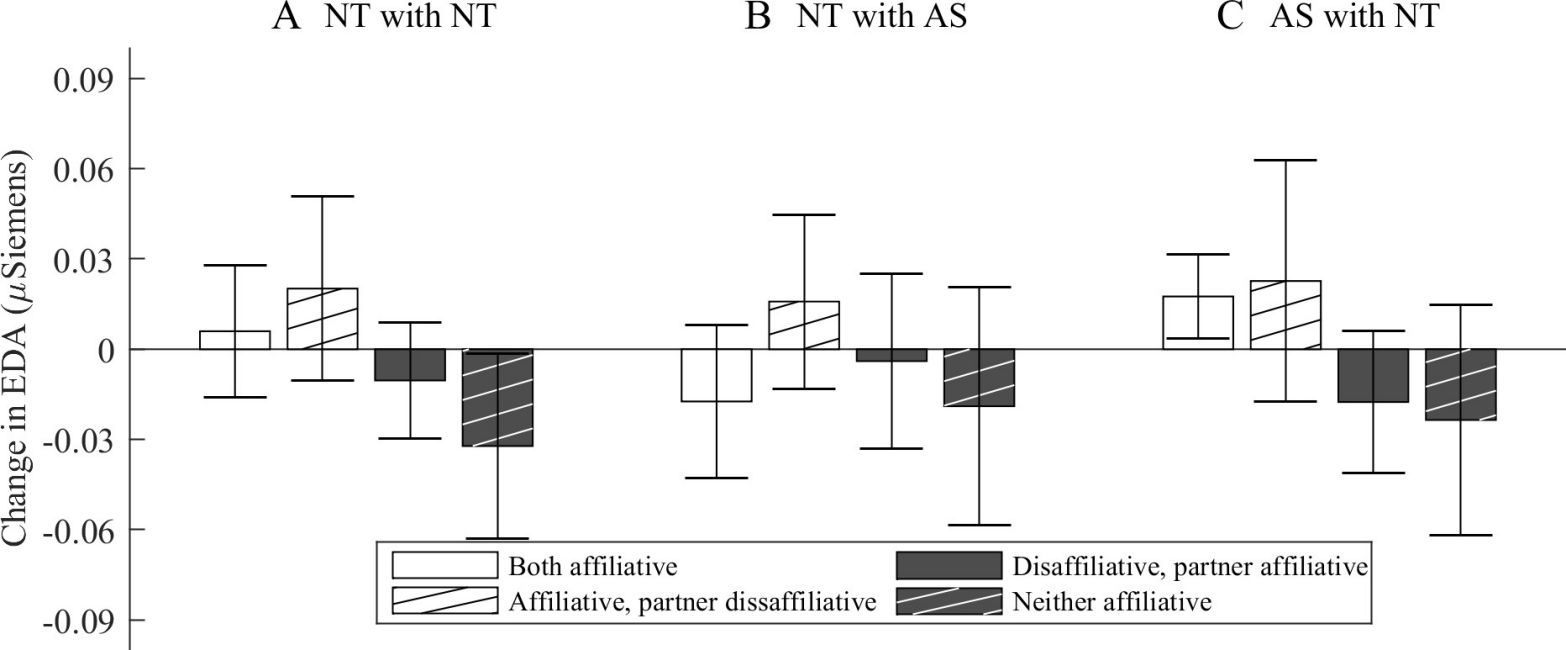
Mean change in EDA compared to previous segment in relation to actors’ and partners’ affiliation (below or above detrended mean) during all segments. Color indicates whether actor affiliation was high (white [white, plain + white, hatched]) or low (dark [dark, plain + dark, hatched]). Texture indicates whether partner affiliation was high (plain [white, plain + dark, plain]) or low (hatched [white, hatched + dark, hatched). Error bars indicate ±2 SEM.

To further illustrate how the actor and partner affiliation interact with physiological response in different dyads, the mean EDA change from one 64-second segment to a next (see Data processing) is illustrated in Fig 2. After individual mean and linear trend was removed, segments with positive values of affiliation were classified as affiliative, and negative values as disaffiliate. EDA change was positive in segments where actor is affiliating (see the white plain and hatched bars in Fig 2, panels A and C), with the exception of NT actors conversing with an AS partner during high actor affiliation (see the white bars in panel B, where the positive and negative effects cancel each other out). However, NT actors, regardless of their partner’s status as an AS or NT participant, exhibited a decreased EDA change brought upon increased partner affiliation (compare the white plain bars in panels A and B to the white hatched ones next to them). Interestingly, this particular partner effect was present only when the NT participants’ own affiliation was high (compare the white bars in Fig 2, panels A and B, to the dark bars [incl. both dark plain and dark hatched bars]). Also, the effect seemed to be stronger in those NT participants who conversed with AS participants (see the large difference in the white bars in panel B). By rough comparison, this partner effect mediates actor effect by more than 0.02 μSiemens. AS participants do not display this mediating effect (see the small difference in the white bars in panel C), but only the general actor effect, as their EDA change was highest when the actor affiliates (white bars in panel C), as compared to when he does not (dark bars in panel C).

## Discussion

To summarize our results, we found several ways in which affiliation influenced the participants’ autonomic nervous system (ANS) responses and their valence expressions. In the NT participants, self-expressed affiliation was associated with heightened HR and also with increased activation of both EMG-OO (smile) and EMG-CS (frown). When interacting with another NT participant, self-expressed affiliation was additionally associated with increased EDA and decreased HRV-HF, indicating increased sympathetic and decreased parasympathetic activation. The EMG responses to self-expressed affiliative behavior were similar independent of the type of dyad, except for the NT participants frowning more during low dominance when discussing with an AS participant. The AS actors did not exhibit any of these patterns. Instead, their self-expressed affiliation was associated with increased EDA during the moments of low dominance. As for valence expressions, AS participants exhibited no significant actor effects. In the NT participants, the experience of partner affiliation was reflected in the decreased levels of EDA (NT/NT dyads) and HR (AS/NT dyads). In the NT/NT dyads, partner affiliation was also associated with increased EMG-OO (smile) and decreased EMG-CS (frown). In contrast, the AS participants responded to their partners’ affiliation with increased EDA and HR. As for valence expressions, AS participants displayed a positive partner effect on EMG-OO (smile).

We will now discuss our results with reference to the specific hypotheses described at the beginning of this paper. In the NT/NT dyads, the results are in line with our hypotheses (1) and (2), and with the earlier results of Peräkylä and colleagues [10], who suggested that a high level of affiliation provided by the listener works to calm down the teller. Our results suggest that, when participants are actively dominating the conversation, a high level of affiliation provided by the partner works to calm them down. While the study of Peräkylä and colleagues [10] focused on the relationship between affiliation and EDA, we showed such response to occur also in HR and HRV-HF. In addition, instead of focusing on the specific moments of storytelling, we showed the pattern to hold for moments of high self-expressed dominance in general (which typically also includes moments of storytelling). Our results on the effect of self-expressed affiliation on the indicators of heightened arousal in the NT/NT dyads are also in line with the suggestion of Peräkylä and colleagues [10] that the giving of affiliation is “hard work.” This result is also in line with our findings about dominance having an overall calming effect on the participants: it is the moments of dominance where a participant is freed from the obligation of tracking the affective import of the co-participants’ speech so as to provide appropriate displays of affiliation—something that can be cognitively challenging [52]. Furthermore, as may be presupposed based on the phenomenon of “emotional contagion” [72], both self-expressed and partner-expressed affiliation were associated with increased amount of smiling. Also our hypotheses for frowning were confirmed: self-expressed affiliation was associated with increased EMG-CS and partner affiliation with decreased EMG-CS.

Regarding our hypothesis (3) that AS participants would experience self-expressed affiliation as particularly taxing, our results are worth attention. While for the NT participants the giving of affiliation was physiologically taxing interactional work, the AS participants did not exhibit such responses. Our EDA results suggest that, for the AS participants, the giving of affiliation is physiologically taxing only when they are *not* dominating the interaction. Thus, even if our hypothesis (3) was not supported by the results, hypothesis (4) that the AS participants’ psychological responses might be reduced when they are dominating the interaction, received partial support. One interpretation of this could be that providing affiliation during moments of their own low dominance might require more conscious effort and social attention from the AS participants in order to actively convey interest and attentiveness towards the NT participant. It is also possible that, when dominating the conversation, the AS participants might have had the possibility to lead the conversation away from those (possibly emotionally salient) topics that they might have experienced as anxiety-provoking. Such conclusions can also be supported by our results on the above-mentioned “calming” effect of dominance being particularly strong in the AS participants, as indicated by the interaction effect of role and dominance for the participants’ heart rate. In this sense, our study is in line with the notion according to which many people with autism spectrum disorders will find particular joy or significance in behaviors typically considered as “autistic” [13, 53], such as talking about their own specific interests. A further study focusing on the content of the AS participants’ talk could shed more specific light on the issue.

Especially exciting are our new findings of behaviors and experiences of NT participants who discuss with an AS participant. Our hypothesis (5) that the displays of affiliation provided by an AS participant will cause higher ANS responses in their NT co-participants was *not* supported by the results. Based on their observational study Hobson and Lee [73, 74] suggested that NT participants might behave differently when discussing with an AS participant, instead of with another NT participant. Such difference has been accounted for with reference to the NT participants’ experience of the “otherness” of the AS persons, which may lead to a certain degree of “uneasiness” [13, 53]. Our results suggest that, even if such a phenomenon might exist on a behavioral level, it does not necessarily exist on the level of physiological responses to partner affiliation. From this point of view, the training of the AS participants’ interactional skills in terms of teaching them to behave in an affiliative way is not in vain—an idea that is, of course, shared by the countless social skill experts and trainers working in the field (see e.g., [75, 76]).

From the point of view of the observations above, our results concerning hypothesis (6) are quite intriguing. According to our results, the displays of affiliation provided by the NT participants are associated with elevated ANS responses in the AS participants. This pattern may be clarified with reference to the AS participants experiencing a high level of affiliation provided by their NT co-participant as challenging—perhaps calling them to stay vigilant in the interaction. In showing that it is the affiliation provided by the NT participant that causes “trouble” in our data, and not the other way round, our results corroborate the bi-directionality of ASD-related interactional problems—the so-called “double empathy problem”—described at the beginning of the paper [22, 23]. It is possible that the amount of emotionally-relevant information conveyed by affiliation from NT participants is somehow “too much” for the participants with AS to cope with, leading to a socio-emotional overflow. This explanation would be in line with the one by Markram and colleagues [77], who characterized autism in terms of hypersensitivity to emotions of others. Consequently, in some cases, emotional expressions by others might then induce anxiety and stress [77, 78]. Another possibility is that the AS participants orient to the moral obligation of reciprocating affiliative expressions, which they might experience as taxing. Such an explanation would be in line with our previous study, where we found that an increased level of affiliation synchrony between NT participants generated a positive interactional experience, as reflected in their post-conversation self-report questionnaires, while affiliation synchrony predicted a more negative interactional experience in the AS participants [62].

Our study on the linkages between ANS responses and interactional affiliation can further our understanding on difficulties of social functioning in young adults with ASD. Deviant physiological responses may reflect the social challenges and the consideration of these responses might prove important for professionals in education and social skill interventions, who seek to increase the social competence of ASD individuals. Measuring treatment efficacy in individuals with ASD has relied primarily on behaviors, with limited attention and evidence to the potential underlying biological mechanisms. Further research could explore more extensively the value of physiological activations during interaction, as they may be useful in measuring treatment efficacy of social skills interventions and educating people with autism spectrum disorders.

Our study has at least seven limitations. First, our sample size is relatively small, which constrains the extent to which we can generalize the results. Second, the methodological approach used in this study did not allow us to control for the content of the participants’ conversations. Even if affiliation and dominance, as general interactional parameters, are as such independent of the content of conversation, it is still possible that topics vary with reference to the relative importance of partner affiliation, which thus might have influenced some of our results. Third, our results may have been impacted by self-selection bias. The sample for this research consists of volunteers socially courageous enough to decide to participate in a study where one is expected to be talking with a stranger. We may thus expect that those who find such situations particularly stressful were not likely to volunteer in our study, which may have influenced our results for the stress-related physiological responses in all our dyads. Fourth, for practical limitations in recruitment, we were unable to include AS/AS dyads in the design of the experiment. Fifth, as this study was based on observer ratings it is possible that some of the actions that were evaluated as affiliative occurred without the participants’ conscious efforts to affiliate, which might have influenced their ANS responses. Sixth, in order to facilitate interpretation of our statistical model, we resorted in categorizing a continuous variable (dominance), which loses information. The results pertaining to dominance have thus less statistical power. Finally, the fact that the participants were strangers to each other may have generated behavioral and physiological response patterns that would be different in interactions between everyday acquaintances, friends, or family members—something that future research should address.

## Conclusions

Our results suggest that a high level of affiliation provided by the conversational partner works to physiologically calm down a participant in interaction—especially, if he at that moment of interaction plays a dominant role in it. At the same time, however, the providing of affiliation is associated with indicators of heightened arousal, which suggest that it is rightly to be considered as interactional “work”, which may even be physiologically taxing. While similar physiological responses to self-expressed affiliation could also be observed in participants with AS when they were not dominating in the conversation, the situation was different at those moments of interaction where the AS participants themselves played the leading role. This finding, together with our results on the AS participants reacting to their partner’s affiliation with an increased level of arousal, points to a fundamental difference in the social meaning of affiliation between the neurotypical participants and the ones with autism spectrum conditions.

## Supporting information

S1 Appendix (DOCX)

S2 Appendix (DOCX)

S1 Demographics (SAV)

S1 Data (SAV)

S1 Syntax (SPS)

## References

[1] Frith U. Autism and Asperger syndrome. Cambridge, UK: Cambridge University Press; 1991.

[2] Szatmari P. Asperger’s syndrome: diagnosis, treatment, and outcome. Ped Clin N Am, 1991;14(1),81–92. 2047334

[3] Smith Myles B, Simpson RL. Asperger syndrome: an overview of characteristics. Focus on Aut and Oth Dev Dis. 2002:17(3),132–7.

[4] Lindström A, Sorjonen ML. Affiliation in conversation In: Sidnell J, Stivers T, editors. The handbook of conversation analysis. Oxford, UK: Wiley-Blackwell; 2012 pp. 350–69.

[5] Stivers T. Stance, alignment, and affiliation during storytelling: when nodding is a token of affiliation. Res Lang Soc Int. 2008;41(1),31–57.

[6] Jefferson G. On the sequential organization of troubles-talk in ordinary conversation. Soc Probl. 1988;35(4),418–41.

[7] Selting M. Affectivity in conversational storytelling: An analysis of displays of anger or indignation in complaint stories. Pragmatics 2010;20(2), 229–277.

[8] Couper-Kuhlen E. Exploring affiliation in the reception of conversational complaint stories In: Peräkylä A, Sorjonen ML, editors. Emotion in interaction. New York, NY: Oxford University Press; 2012 pp 113–46.

[9] Peräkylä A, Ruusuvuori J. Facial expression and interactional regulation of emotion In: Peräkylä A, Sorjonen ML, editors. Emotion in interaction. New York, NY: Oxford University Press; 2012 pp. 64–91.

[10] Peräkylä A, Henttonen P, Voutilainen L, Kahri M, Stevanovic M, Sams M, Ravaja M. Sharing the emotional load: recipient affiliation calms down the storyteller. Soc Psych Quart. 2015;78(4):301–23.

[11] Hochschild A. Emotion work, feeling rules, and social structure. Am J Sociol. 1979;85(3),551–75.

[12] Hochschild A. The managed heart: Commercialization of human feeling. Berkeley: University of California Press; 1983.

[13] De Jaegher H. Embodiment and sense-making in autism. Front Integr Neurosci. 2013;26. 10.3389/fnint.2013.00015 PMC3607806 23532205

[14] Baron-Cohen S, Leslie AM, Frith U. Does the autistic child have a theory of mind? Cognition 1985;21, 37–46. 2934210 10.1016/0010-0277(85)90022-8

[15] Senju A, Southgate V, White S, Frith U. Mindblind eyes: an absence of spontaneous theory of mind in Asperger syndrome. Science 2009;325(5942):883–5. 10.1126/science.1176170 19608858

[16] Pillai D, Sheppard E, Ropar D, Marsh L, Pearson A, Mitchell P. Using other minds as a window onto the world: guessing what happened from clues in behaviour. J Autism Dev Disord. 2014;44(10):2430–9. 10.1007/s10803-014-2106-x 24710812

[17] Cassidy S, Ropar D, Mitchell P, Chapman P. Can adults with autism spectrum disorders infer what happened to someone from their emotional response? Autism Res. 2014;7(1):112–23 10.1002/aur.1351 24307231

[18] Cassidy S, Mitchell P, Chapman P, Ropar D. Processing of spontaneous emotional responses in adolescents and adults with autism spectrum disorders: effect of stimulus type. Autism Res. 2015;8(5):534–44. 10.1002/aur.1468 25735657 PMC4964927

[19] Fletcher-Watson S, Leekam SR, Benson V, Frank MC, Findlay JM. Eye-movements reveal attention to social information in autism spectrum disorder. Neuropsychologia. 2009;47(1):248–57. 10.1016/j.neuropsychologia.2008.07.016 18706434

[20] Freeth M, Chapman P, Ropar D, Mitchell P. Do gaze cues in complex scenes capture and direct the attention of high functioning adolescents with ASD? Evidence from eye-tracking. J Autism Dev Disord. 2010;40(5):534–47. 10.1007/s10803-009-0893-2 19904597

[21] Doherty-Sneddon G, Whittle L, Riby DM. Gaze aversion during social style interactions in autism spectrum disorder and Williams syndrome. Res Dev Disabil. 2013;34(1):616–26. 10.1016/j.ridd.2012.09.022 23123875

[22] Sheppard E, Pillai D, Wong GT, Ropar D, Mitchell P. How easy is it to read the minds of people with autism spectrum disorder? J Autism Dev Disord. 2016;46(4):1247–54. 10.1007/s10803-015-2662-8 26603886

[23] Milton D. On the ontological status of autism: the ‘double empathy problem’, Disability & Society. 2012;27:6:883–87.

[24] Karemaker JM. An introduction into autonomic nervous function. Physiol Meas. 2017;38(5),R89 10.1088/1361-6579/aa6782 28304283

[25] Laborde S, Mosley E, Thayer JF. Heart rate variability and cardiac vagal tone in psychophysiological research–recommendations for experiment planning, data analysis, and data reporting. Front Psychol. 2017;8,213 10.3389/fpsyg.2017.00213 28265249 PMC5316555

[26] Saghir H, Dupuis A, Chau T, Kushki A. Atypical autonomic nervous system complexity accompanies social cognition task performance in ASD. Res Aut Spect Dis, 2017;39(Supplement C),54–62.

[27] Kushki A, Drumm E, Pla Mobarak M, Tanel N, Dupuis A, Chau T, et al Investigating the autonomic nervous system response to anxiety in children with autism spectrum disorders. PLoS ONE. 2013;8(4),e59730 10.1371/journal.pone.0059730 23577072 PMC3618324

[28] Ming X, Patel R, Kang V, Chokroverty S, Julu PO. Respiratory and autonomic dysfunction in children with autism spectrum disorders. Brain Dev. 2016;38(2),225–32. 10.1016/j.braindev.2015.07.003 26235973

[29] Jansen LMC, Gispen-De Wied CC, Wiegant VM, Westenberg HGM, Lahuis BE, Van Engeland H. Autonomic and neuroendocrine responses to a psychosocial stressor in adults with autistic spectrum disorder. J Aut Dev Dis. 2006;36(7),891–99. 10.1007/s10803-006-0124-z 16865550

[30] Smeekens I, Didden R, Verhoeven EWM. Exploring the relationship of autonomic and endocrine activity with social functioning in adults with autism spectrum disorders. J Aut Dev Dis, 2015;45(2),495–505. 10.1007/s10803-013-1947-z 24062183

[31] Porges SW, Macellaio M, Stanfill SD, McCue K, Lewis GF, Harden ER, et al Respiratory sinus arrhythmia and auditory processing in autism: modifiable deficits of an integrated social engagement system? Int J Psychophy. 2013;88(3),261–70. 10.1016/j.ijpsycho.2012.11.009 PMC3610863 23201146

[32] Neuhaus E, Bernier R, Beauchaine TP. Brief report: social skills, internalizing and externalizing symptoms, and respiratory sinus arrhythmia in autism. J Aut Dev Dis. 2014;44(3),730–7. 10.1007/s10803-013-1923-7 23982488

[33] Harder R., Malow BA, Goodpaster RL, Iqbal F, Halbower A, Goldman SE, et al Heart rate variability during sleep in children with autism spectrum disorder. Clin Auton Res. 2016;26(6):423–32. 10.1007/s10286-016-0375-5 27491489 PMC5106315

[34] Mathersul D, McDonald S, Rushby JA. Automatic facial responses to affective stimuli in high-functioning adults with autism spectrum disorder. Physiol Beh, 2013a;109,14–22. 10.1016/j.physbeh.2012.10.008 23142408

[35] Mathersul D, McDonald S, Rushby JA. Psychophysiological correlates of social judgement in high-functioning adults with autism spectrum disorder. Int J Psychophy, 2013b;87(1),88–94. 10.1016/j.ijpsycho.2012.11.005 23183316

[36] Hubert BE, Wicker B, Monfardini E, Deruelle C. Electrodermal reactivity to emotion processing in adults with autistic spectrum disorders. Aut. 2009;13(1),9–19. 10.1177/1362361308091649 19176574

[37] Kylliäinen A. Hietanen JK. Skin conductance responses to another person’s gaze in children with autism. J Aut Dev Dis. 2006;36(4),517–25. 10.1007/s10803-006-0091-4 16555137

[38] Kaartinen M, Puura K, Mäkelä T, Rannisto M, Lemponen R, Helminen M, et al Autonomic arousal to direct gaze correlates with social impairments among children with ASD. J Aut Dev Dis. 2012;42(9),1917–27. 10.1007/s10803-011-1435-2 22215435

[39] O’Haire M, Mckenzie S, Beck A, Slaughter V. Animals may act as social buffers: skin conductance arousal in children with autism spectrum disorder in a social context. Dev Psychol. 2015;57(5),584–95. 10.1002/dev.21310 PMC8447904 25913902

[40] Sullivan HS. The interpersonal theory of psychiatry. New York: Norton; 1953.

[41] Leary T. Interpersonal diagnosis of personality. New York: Ronald Press; 1957.

[42] Carson RC. Interaction concepts of personality. Chicago, IL: Aldine; 1969.

[43] Kiesler DJ. The 1982 Interpersonal Circle: a taxonomy for complementarity in human transactions. Psychol Rev. 1983;90(3),185–214.

[44] Kiesler DJ. Contemporary interpersonal theory and research: Personality, psychopathology, and psychotherapy New York, NY: Wiley; 1996.

[45] Wiggins JS. Circumplex models of interpersonal behavior in clinical psychology In: Kendall PC, Butcher JN, editors. Handbook of research methods in clinical psychology. New York: Wiley; 1982 pp. 183–221.

[46] Widiger TA. Personality, interpersonal circumplex, and DSM-5: a commentary on five studies. J Pers Assess. 2010;92,528–32. 10.1080/00223891.2010.513707 20954054

[47] Yaughn E, Nowicki S. Close relationships and complementary interpersonal styles among men and women. J Soc Psychol. 1999;139,473–8.

[48] Gurtman MB. Interpersonal complementarity: Integrating interpersonal measurement with interpersonal models. J Coun Psychol. 2001;48(1):97–110.

[49] Smith JL, Ruiz JM. Interpersonal orientation in context: correlates and effects of interpersonal complementarity on subjective and cardiovascular experiences. J Pers, 2007;75,679–708. 10.1111/j.1467-6494.2007.00453.x 17576355

[50] Sadler P, Ethier N, Gunn GR, Duong D, Woody E. Are we on the same wavelength? Complementarity as shared cyclical patterns within an interaction. J Pers Soc Psychol. 2009;97,1005–20. 10.1037/a0016232 19968416

[51] Lizdek I, Sadler P, Woody E, Ethier N, & Malet G. Capturing the stream of behavior: a computer-joystick method for coding interpersonal behavior continuously over time. Soc Sci Comp Rev. 2012;30,513–21.

[52] Voutilainen L, Henttonen P, Kahri M, Kivioja M, Ravaja N, Sams M et al Affective stance, ambivalence, and psychophysiological responses during conversational storytelling. Journal of Pragmatics. 2012;68:1–24.

[53] Attwood T. Asperger’s syndrome: A guide for parents and professionals. London: Kingsley; 1998.

[54] World Health Organization. ICD-10, the ICD-10 classification of mental and behavioural disorders: Diagnostic criteria for research Geneva: World Health Organization; 1993.

[55] Baron-Cohen S, Wheelwright S, Skinner R, Martin J, Clubley E. The autism-spectrum quotient (AQ): evidence from asperger syndrome/high-functioning autism, males and females, scientists and mathematicians. J Aut Dev Dis. 2001;31(1):5–17. 10.1023/a:1005653411471 11439754

[56] Baron-Cohen S, Wheelwright S. The empathy quotient: an investigation of adults with Asperger syndrome or high functioning autism, and normal sex differences. J Aut Dev Dis. 2004;34(2):163–75. 10.1023/b:jadd.0000022607.19833.00 15162935

[57] Baron-Cohen S, Richler J, Bisarya D, Gurunathan N, Wheelwright S. The systemizing quotient: an investigation of adults with Asperger syndrome or high–functioning autism, and normal sex differences. Phil Trans R Soc Lond B Biol Sci. 2003;358(1430):361–74. 12639333 10.1098/rstb.2002.1206 PMC1693117

[58] Wechsler D. Wechsler Adult Intelligence Scale–Revised (WAIS-R). New York: The Psychological Corporation; 1981. Finnish translation. Translated and adapted by permission. Helsinki: Psykologien Kustannus Oy; 1992.

[59] Berntson GG, Quigley KS, Lozano D. Cardiovascular psychophysiology In: Cacioppo JT, Tassinary LG, Berntson GG, editors. Handbook of psychophysiology. 3rd ed Cambridge: Cambridge University Press; 2007 pp. 182–210.

[60] Malik M. Heart rate variability. Standards of measurement, physiological interpretation, and clinical use. Task Force of the European Society of Cardiology and the North American Society of Pacing and Electrophysiology. Eur. Heart J. 1996;17:354–81. 8737210

[61] Sadler P, Woody E McDonald K, Lizdek I, Little J. A lot can happen in a few minutes: examining dynamic patterns within an interaction to illuminate the interpersonal nature of personality disorders. J Pers Dis. 2015;29(4),526–46. 10.1521/pedi.2015.29.4.526 26200850

[62] Stevanovic M, Henttonen P, Koski S, Kahri M, Voutilainen L, Koskinen E, et al On the Asperger experience of interaction: interpersonal dynamics in dyadic conversations. J Aut. 2017;4(2). 10.7243/2054-992X-4-2

[63] Brainard DH, Vision S. The psychophysics toolbox. Spat Vis. 1997;10(4):433–6. 9176952

[64] Pelli DG. The VideoToolbox software for visual psychophysics: transforming numbers into movies. Spat Vis. 1997;10(4),437–42. 9176953

[65] Kleiner M, Brainard D, Pelli D, Ingling A, Murray R, Broussard C. What’s new in psychtoolbox-3. Perception. 2007;36(14):1–16.

[66] Cronbach LJ. Coefficient alpha and the internal structure of tests. Psychometrika. 1951;16(3):297–334.

[67] Nunnally JC, Bernstein I. Psychometric theory. 3rd ed New York, NY: McGraw-Hill; 1994.

[68] George D, Mallery P. SPSS for Windows step by step: A simple guide and reference. 11.0 update. 4th ed Boston: Allyn & Bacon; 2003.

[69] De Carvalho JL, Da Rocha AF, de Oliveira Nascimento FA, Neto JS, Junqueira LF. Development of a Matlab software for analysis of heart rate variability. In: Proceedings of the 6th IEEE International conference on signal processing, 2002; Beijing, China. pp. 1488–91.

[70] Kenny DA, Kashy DA, Cook WL, Simpson JA. Dyadic data analysis. Methodology in the social sciences. New York, NY: Guilford; 2006.

[71] Benjamini Y, Hochberg Y. Controlling the false discovery rate: a practical and powerful approach to multiple testing. J R Stat Soc Series B Stat Methodol. 1995;57(1):289–300.

[72] Hatfield E, Cacioppo JT, Rapson RL. Emotional contagion. Cambridge, UK:CUP; 1994.

[73] Hobson RP, Lee A. Hello and goodbye: a study of social engagement in autism. J Aut Dev Dis. 1998;28(2),117–27. 10.1023/a:1026088531558 9586774

[74] Hobson RP. The cradle of thought. London: Macmillan; 2002.

[75] Gutstein SE. Autism/Aspergers: Solving the Relationship Puzzle Arlington, TX: Future Horizons; 2001.

[76] Ke F, Whalon K, Yun J. Social skill interventions for youth and adults with autism spectrum disorder: a systematic review. Rev Ed Res. 2018;88(1),3–42.

[77] Markram H, Rinaldi T, Markram K. The intense world syndrome–an alternative hypothesis for autism. Front Neur. 2007;1(1),77–96. 10.3389/neuro.01.1.1.006.2007 PMC2518049 18982120

[78] Markram K, Markram H. The intense world theory–a unifying theory of the neurobiology of autism. Front Neur. 2010;4:224. 10.3389/fnhum.2010.00224 PMC3010743 21191475

