# Supplementary material for: Physiological responses to affiliation during conversation: Comparing neurotypical males and males with Asperger syndrome
Source: PLoS One. 2019 Sep 18;14(9):e0222084. doi: 10.1371/journal.pone.0222084 (PMC6750568; doi:10.1371/journal.pone.0222084)
Supplement: S1 Appendix — (DOCX) [file pone.0222084.s001.docx]

**S1 Appendix: Additional figures**

Figures S1-S7 represent model estimated means of dependent physiological variables during low (-1SD) and high (+1SD) actor and partner affiliation.

**
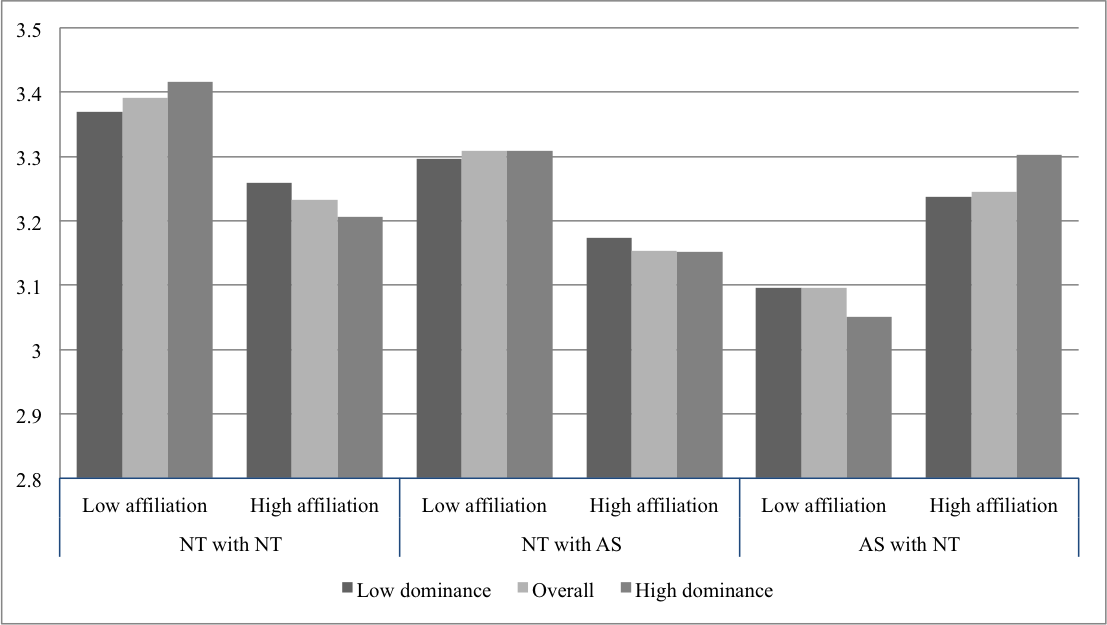
**

Figure S1: Relation of partner affiliation to EDA in all dyads. Values are estimated at -1SD and +1SD points of partner affiliation. Values on y axis represent µSiemens.


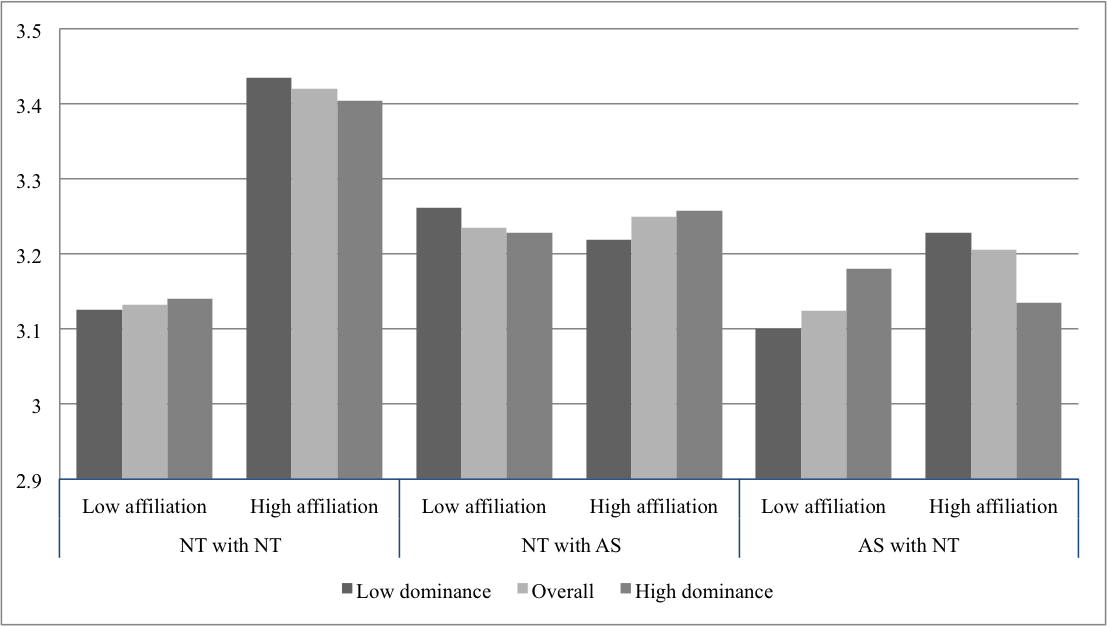


Figure S2: Relation of actor affiliation to EDA in all dyads. Values are estimated at -1SD and +1SD points of actor affiliation. Values on y axis represent µSiemens.


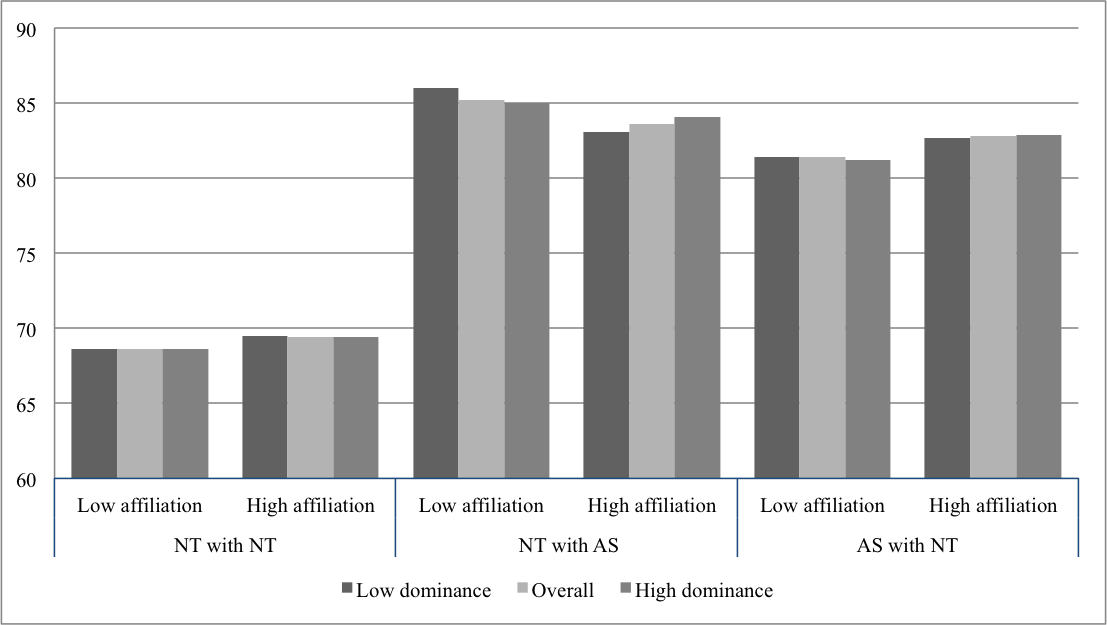


Figure S3: Relation of partner affiliation to HR in all dyads. Values are estimated at -1SD and +1SD points of partner affiliation. Values on y axis represent bpm (beats per minute).


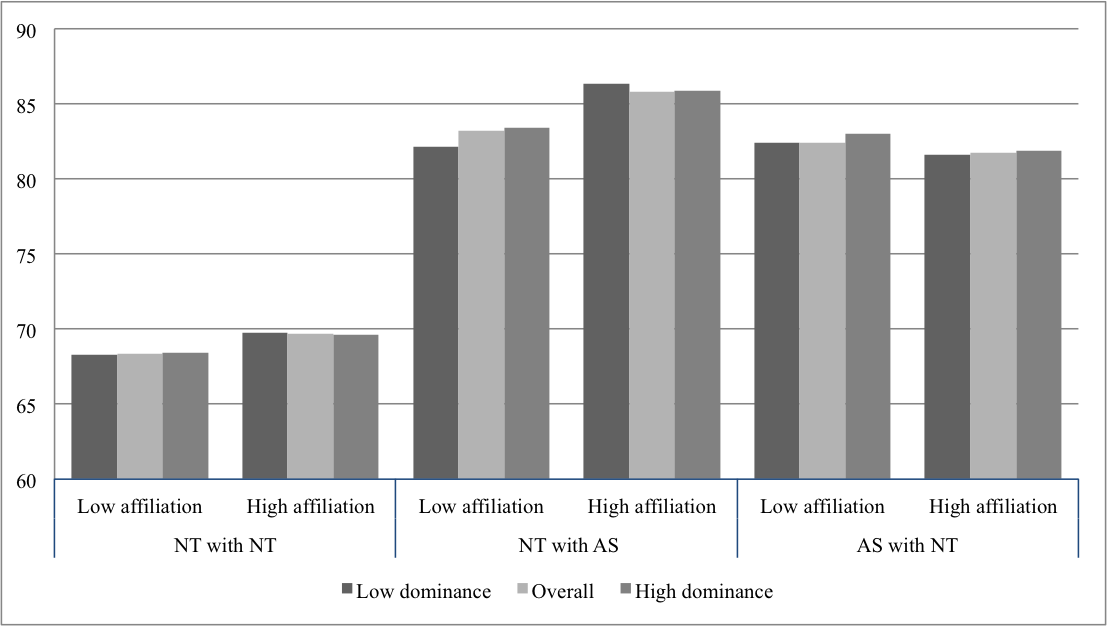


Figure S4: Relation of actor affiliation to HR in all dyads. Values are estimated at -1SD and +1SD points of actor affiliation. Values on y axis represent bpm (beats per minute).


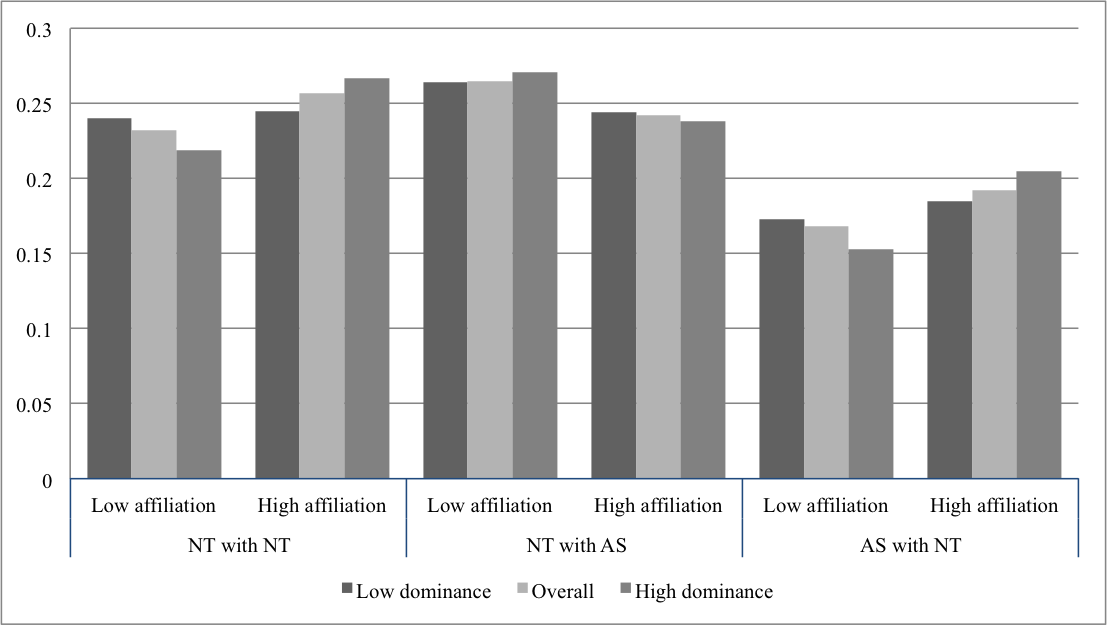


Figure S5: Relation of partner affiliation to HRV-HF in all dyads. Values are estimated at -1SD and +1SD points of partner affiliation. Values on y axis represent normalized portion of high frequency (0.15-0.4 Hz) component of HRV.


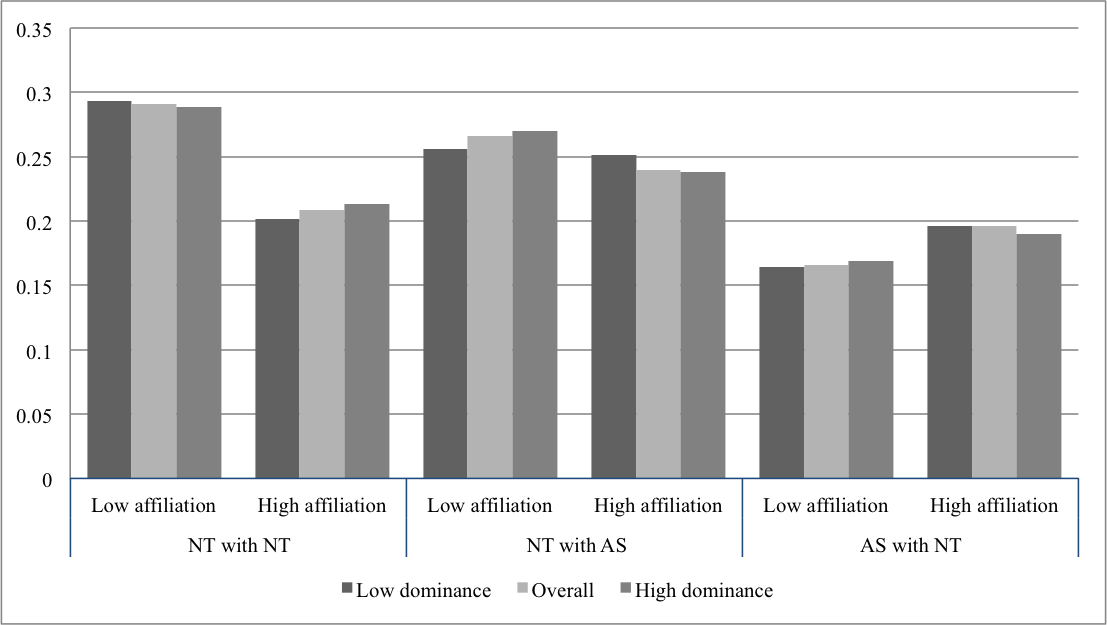


Figure S6: Relation of actor affiliation to HRV-HF in all dyads. Values are estimated at -1SD and +1SD points of actor affiliation.Values on y axis represent normalized portion of high frequency (0.15-0.4 Hz) component of HRV.


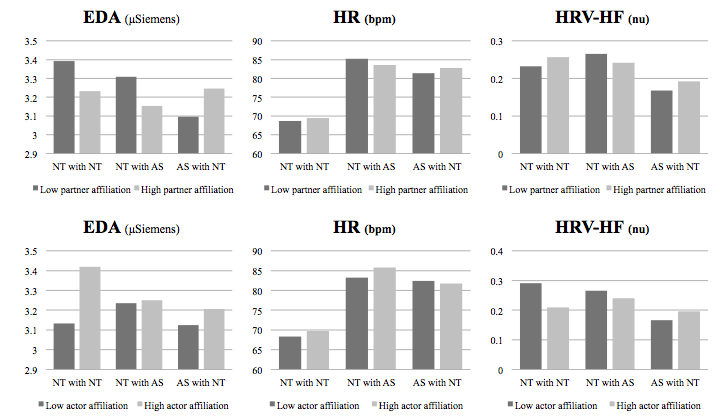


Figure S7. Relation of actor and partner effects of affiliation with physiological dependent variables in all dyads. Values are estimated at -1SD (low) and +1SD (high) points of partner and actor effects.
