# Supplementary material for: Physiological responses to affiliation during conversation: Comparing neurotypical males and males with Asperger syndrome
Source: PLoS One. 2019 Sep 18;14(9):e0222084. doi: 10.1371/journal.pone.0222084 (PMC6750568; doi:10.1371/journal.pone.0222084)
Supplement: S2 Appendix — (DOCX) [file pone.0222084.s002.docx]

**S2 Appendix: Joystick practice**

The observers heard one-sentence-long verbal descriptions of interactional events, in response to which they needed to move the joystick to the correct location in the Cartesian plane.

DOMINANCE

4 20 3 17 9

14 12

2 14

18 8

5 21

11 6

13 16

1 7

10 19

AFFILIATION

Figure S8: Descriptions of interactional events and their location in the Cartesian plane representing the interpersonal circumplex.

1. The participant listens to his/her co-participant’s speech while staring at his/her own shoes.

2. The participant laments about the idiocy of other people.

3. The participant determines the topic to be talked about next.

4. The participant expresses disagreement with his/her co-participant.

5. The participant undermines the significance of what his/her co-participant has just told him/her.

6. The participant provides friendly and encouraging backchannelling responses to his/her co-participant’s telling.

7. The participant refrains from expressing his/her opinion on the topic of discussion.

8. The participant initiates a new topic in anticipation of an embarrassing silence.

9. The participant claims to understand for his/her co-participant’s views, even if s/he disagrees.

10. The participant acts shy and scared.

11. The participant seems indifferent towards his/her co-participant.

12. The participant expresses strong agreement with his/her co-participant.

13. The participant seems to be bored with the discussion.

14. The participant interrupts his/her co-participant’s telling with a telling of his/her own.

15. The participant encourages his/her co-participant go on talking by asking specifying questions.

16. The participant agrees with his/her co-participant minimally, but does not further the topic.

17. The participant tells funny stories about his/her fooleries.

18. The participant talks arrogantly about him/herself.

19. The participant listens expressionlessly to his/her co-participant’s speech.

20. The participant poses challenging questions.

21. The participant listens to his/her co-participant’s telling in an active and sympathizing way.
